# Supplementary material for: Improved prediction of solvation free energies by machine-learning polarizable continuum solvation model
Source: Nat Commun. 2021 Jun 18;12:3584. doi: 10.1038/s41467-021-23724-6 (PMC8213834; doi:10.1038/s41467-021-23724-6)
Supplement: Supplementary file 2 — Description of Additional Supplementary Files [file 41467_2021_23724_MOESM2_ESM.pdf]

Description of Additional Supplementary Files

File Name: Supplementary Data 1
Description: Full list of the studied samples

File Name: Supplementary Software 1
Description: C++ code for implementing the proposed methods
