# Supplementary material for: Improved prediction of solvation free energies by machine-learning polarizable continuum solvation model
Source: Nat Commun. 2021 Jun 18;12:3584. doi: 10.1038/s41467-021-23724-6 (PMC8213834; doi:10.1038/s41467-021-23724-6)
Supplement: Supplementary file 4 — Supplementary Software 1 [file 41467_2021_23724_MOESM4_ESM.zip › C++/user_instructions.pdf]

User instructions for applying the developed models using the provided C++ code

This document is provided as a tutorial for readers who are interested in implementing the developed methods. In case of observing any bug or issue, please feel free to contact the authors (alibakhshi@pctc.uni-kiel.de) or (hartke@pctc.uni-kiel.de).

In this tutorial we demonstrate how to generate the molecular representations required by different models via the Gaussian quantum-chemistry software and how to generate free-energy predictions from this model, with the provided piece of software. Although we use Gaussian quantum-chemistry software in the present tutorial, the other standard software packages also commonly calculate and print the same representations, probably under a slightly different terminology.

We developed a total number of 4 different models, which are distinguished by the applied computational level of theory and model inputs (see the manuscript for details).

The main steps towards predicting solvation free energy via the developed models are summarized as follows:

1- After selecting the model of choice for predicting solvation free energy, the required model inputs should be acquired based on the variable IDs specified in tables 1 and 2.
   If you use the Gaussian software, examples of input lines for different solvation models are provided in appendix I.
2- In the next step, the user needs to prepare "key.txt" and "input.txt" files. The file key.txt should have only one line and two space-separated numbers, otherwise an error message will be generated. The first number is the model ID as specified in first column of table 1. The second number is the number of samples available in the "input.txt" file for which the solvation free energy should be calculated.
   In the input.txt file, each line corresponds to one sample, and the different columns, which are separated by one or more spaces (it is format-free), should represent the required model input variable, in the same order as specified in table 1.
3- The created key.txt, input.txt and the C++ code dependencies (i.e. FE_predictor.cpp and settings.ini) should be placed in the same directory. After that, the C++ code should be compiled and run as explained in the following.

**Table 1- specifications of the developed models**														
**model ID**	**Solvation model**	**method**	**input variable IDs**											
1	$CPCM_{x=0.5}$	B3LYP/6-31G*	2	3	4	6	7	9	12	14	15	16		
2	$CPCM_{x=0.5}$	DSD-PBEP86-D3/def2TZVP	1	2	3	4	7	8	9	10	11	14	16	
3	COSMO-RS/ $CPCM_{x=0.5}$	B3LYP/6-31G*	1	7	8	9	11	12	14	15	16			
4	COSMO-RS/ CPCM	DSD-PBEP86-D3/def2TZVP	1	2	3	7	9	10	11	12	15	16		

For models 3 and 4, the SCRF energy components (inputs with IDs 2-15) are computed via $CPCM_{x=0.5}$ and CPCM solvation model while the continuum solvation model predicted solvation free energy is computed via COSMO-RS model. For model 1 and 2 all model inputs are computed for via $CPCM_{x=0.5}$ solvation model. Details of input variable IDs are specified in table 2.

variable ID	Physical interpretation	Corresponding term in Gaussian output
1	cf. the same ID in table 1 in manuscript	"DeltaG (solv)"
2	cf. the same ID in table 1 in manuscript	"<psi(0)| H |psi(0)>"
3	cf. the same ID in table 1 in manuscript	"<psi(0)|H+V(0)/2|psi(0)> "
4	cf. the same ID in table 1 in manuscript	" <psi(0)|H+V(f)/2|psi(0)>"
5	cf. the same ID in table 1 in manuscript	"<psi(f)| H |psi(f)>"
6	cf. the same ID in table 1 in manuscript	"<psi(f)|H+V(f)/2|psi(f)>"
7	cf. the same ID in table 1 in manuscript	"(Unpolarized solute)-Solvent"
8	cf. the same ID in table 1 in manuscript	"(Polarized solute)-Solvent"
9	cf. the same ID in table 1 in manuscript	"Solute polarization"
10	cf. the same ID in table 1 in manuscript	"Total electrostatic"
11	cf. the same ID in table 1 in manuscript	"Cavity surface area"
12	cf. the same ID in table 1 in manuscript	"Cavity volume"
13	cf. the same ID in table 1 in manuscript	"KE"
14	cf. the same ID in table 1 in manuscript	"PE"
15	cf. the same ID in table 1 in manuscript	"EE"
16	dielectric constant of solvent	

<u>For Linux users:</u>

With a C++-compiler like g++ available, first compile the provided C++ program. For this, open a terminal, move to the directory containing the required files, and run the one-line command

g++ FE_predictor.cpp –o SFE

The resulting compiled program can then be invoked with

./ SFE

This generates a new text file named “Predicted_SolvationFE.txt”, containing the predicated solvation free energies of the given samples, in the same order as in the input.txt file.

<u>For windows users:</u>
An easy implementation can be done by installing the code::blocks software with mingw included (we tested codeblocks-20.03mingw-32bit-setup.exe) and after installation and running, go through
File → new → project → console application → go → C++ → defining a name and path → Finish
Then move the input.txt, key.txt and settings.ini files to the directory specified previously in code::blocks, open the source → main.cpp in the left-side menu and replace all the contents of the main window with the contents of the FE_predictor.cpp file. Then simply push the build and run buttons to generate the results.

For many of the user-sided potential sources of error found in the input or key files, an error message informing about possible reasons and solutions will be generated. For other cases, you are welcome to contact us.

Appendix A: DEMO

In this demo we calculate solvation free energy for 50 solute-solvent mixtures listed in ‘DEMO_samples.xlsx’ in demo directory via the model defined in table 1 with ID 1. The experimentally determined solvation free energies for these samples taken from the Minnesota solvation database are listed in FE_exp.txt.

For each one of the samples, we carried out QM computations in Gaussian as explained in Appendix B. As specified in table 1, for the selected model, the variables with IDs 2, 3, 4, 6, 7, 9, 12, 14, 15 and 16 need to be acquired based on the specifications of each variable ID clarified in table 2 for each sample from the Gaussian log file. The final prepared input file is available in the demo directory. As you can see in the input.txt file, we have 50 lines (each one for each sample) and for each line the calculated variables with IDs 2, 3, 4, 6, 7, 9, 12, 14, 15 and 16 are listed in the subsequent columns respectively.

We also need to create a ket.txt file with two numbers, the first one is the model ID which for this example is 1 and the second one is the number of samples (50 for this example). After these two files created, we only need to compile and rum the C++ code as explained in the previous section. After seeing the “Computations successfully finished!” message, the predicted solvation free energies can be found in Predicted_SolvationFE.txt text file in the same directory. For this example, AAD of 0.4174 kcal/mol is obtained for the studied samples which can be calculated also using the ‘calculate_MUE.m’ matlab file in the DEMO directory.

Appendix B: The example input file to run the Gaussian computations

After optimizing the structure of the solute, generating the required macroscopic observables can be achieved by putting the following commands in the Gaussian input file (here the example of an unknown solute and water solvent and computations for B3lyp/6-31G* level of theory is provided).

IEF-PCM solvation model:

```
#P b3lyp /6-31G* SCRF=(PCM,Solvent=Water,externaliteration,dovacuum) nosymm
SCF=(MaxCycle=30,tight) int=finegrid
```

The Eps in the last line, which comes after the specifications of the molecules, is the corrected dielectric constant calculated via equation 5 in the manuscript.

CPCM solvation model:

```
#P b3lyp/6-31G* SCRF=(CPCM,Solvent=$line1,externaliteration,dovacuum) nosymm
SCF=(MaxCycle=30,tight) int=finegrid
```

CPCMx=0.5 solvation model:

```
#P b3lyp/6-31G* SCRF=(CPCM,Solvent=Water,externaliteration,dovacuum,Read)
nosymm SCF=(MaxCycle=30,tight) int=finegrid

Molecule specifications (charge and spin multiplicity, xyz positions,…)

Eps=52.5702
```

The Eps in the last line, which comes after the specifications of the molecules, is the corrected dielectric constant calculated via equation 5 in the manuscript.
